# Roles of Ion and Water Channels in the Cell Death and Survival of Upper Gastrointestinal Tract Cancers

**DOI:** 10.3389/fcell.2021.616933

**Published:** 2021-03-11

**Authors:** Atsushi Shiozaki, Yoshinori Marunaka, Eigo Otsuji

**Affiliations:** ^1^Division of Digestive Surgery, Department of Surgery, Kyoto Prefectural University of Medicine, Kyoto, Japan; ^2^Department of Molecular Cell Physiology, Graduate School of Medical Science, Kyoto Prefectural University of Medicine, Kyoto, Japan; ^3^Research Institute for Clinical Physiology, Kyoto Industrial Health Association, Kyoto, Japan; ^4^Research Center for Drug Discovery and Pharmaceutical Development Science, Research Organization of Science and Technology, Ritsumeikan University, Kusatsu, Japan

**Keywords:** esophageal cancer, gastric cancer, ion channels, water channels, intracellular pH

## Abstract

Ion and water channels were recently shown to be involved in cancer cell functions, and various transporter types have been detected in upper gastrointestinal tract (UGI) cancers. Current information on the expression and roles of these channels and transporters in the death and survival of UGI cancer cells was reviewed herein, and the potential of their regulation for cancer management was investigated. Esophageal cancer (EC) and gastric cancer (GC) cells and tissues express many different types of ion channels, including voltage-gated K^+^, Cl^–^, and Ca^2+^, and transient receptor potential (TRP) channels, which regulate the progression of cancer. Aquaporin (AQP) 1, 3, and 5 are water channels that contribute to the progression of esophageal squamous cell carcinoma (ESCC) and GC. Intracellular pH regulators, including the anion exchanger (AE), sodium hydrogen exchanger (NHE), and vacuolar H^+^-ATPases (V-ATPase), also play roles in the functions of UGI cancer cells. We have previously conducted gene expression profiling and revealed that the regulatory mechanisms underlying apoptosis in ESCC cells involved various types of Cl^–^ channels, Ca^2+^ channels, water channels, and pH regulators (Shimizu et al., 2014; Ariyoshi et al., 2017; Shiozaki et al., 2017, 2018a; Kobayashi et al., 2018; Yamazato et al., 2018; Konishi et al., 2019; Kudou et al., 2019; Katsurahara et al., 2020, 2021; Matsumoto et al., 2021; Mitsuda et al., 2021). We have also previously demonstrated the clinicopathological and prognostic significance of their expression in ESCC patients, and shown that their pharmacological blockage and gene silencing had an impact on carcinogenesis, indicating their potential as targets for the treatment of UGI cancers. A more detailed understanding of the molecular regulatory mechanisms underlying cell death and survival of UGI cancers may result in the application of cellular physiological methods as novel therapeutic approaches.

## Introduction

Upper gastrointestinal tract (UGI) cancers, such as esophageal cancer (EC) and gastric cancer (GC), have high recurrence rates and are one of the leading causes of cancer-related death globally (Kamangar et al., 2006; Brenner et al., 2009; Hartgrink et al., 2009). EC is the ninth most common cancer and the sixth most common cause of cancer deaths worldwide, and GC is the sixth most common cancer and the third most common cause of cancer deaths (Bray et al., 2018). The vast majority of ECs are either esophageal squamous cell carcinoma (ESCC) or esophageal adenocarcinomas (EAC). Although the prognosis of patients with UGI cancers has recently been improved by advances in surgical techniques, chemotherapy, radiotherapy, immunotherapy, and molecular targeted therapy (Jemal et al., 2011; Udagawa et al., 2012; Nashimoto et al., 2013; Tachimori et al., 2019), they remain poor, particularly for patients with advanced disease. Limited information is currently available on the efficacy of potential drug combinations against EC and GC because of the ability of these cells to evade apoptosis. Therefore, further studies on the molecular mechanisms regulating the cell death and survival of UGI cancers are needed for the development of more effective treatments.

Ion and water channels/transporters have important roles in cellular functions. Their physiological contribution to cell death and survival is crucial because cell volume changes, which require the movement of ions and water molecules across cell membranes, are critical for apoptosis (Okada et al., 2001, 2006). The involvement of ion and water channels in cancer cell functions was recently demonstrated, and thus, their regulation has potential as a novel strategy in cancer therapies (Pedersen and Stock, 2013; Pardo and Stuhmer, 2014; Shiozaki et al., 2014; Lastraioli et al., 2015; Nagaraju et al., 2016; Xia et al., 2017; Anderson et al., 2019).

Current information on the functions of ion and water channels in the cell death and survival of UGI cancers was systematically reviewed herein. The main aim of this review was to investigate the potential of cellular physiological strategies, such as the regulation of ion channels, water channels, and pH regulators, in the clinical management of EC and GC.

### Potassium (K^+^) Channels

Several subtypes of K^+^ channels were recently shown to be expressed in human EC and GC cells and play important roles in cell death and survival (Table 1). The expression of several voltage-gated K^+^ channels (Kv) is modified in UGI cancers. Han et al. (2007) reported that the up-regulated expression of potassium voltage-gated channel, shaker-related subfamily, member 5 (KCNA5), also known as Kv1.5, increased K^+^ current density as well as GC cell sensitivity to multiple chemotherapeutic drugs by regulating drug-induced apoptosis. Furthermore, a component of the delayed rectifier K^+^ current was found to be encoded by the human ether-a-go-go-related gene (HERG). The expression of HERG channels was shown to be limited in GC, and the HERG channel blocker, cisapride, and genetic knockdown by small interfering RNA (siRNA) technology induced apoptosis (Shao et al., 2005, 2008). Zhang et al. (2012) demonstrated that the expression of HERG was crucial for the cisplatin-mediated induction of apoptosis in human GC. However, in these studies, molecular mechanisms of the regulation of apoptosis via HERG had not been shown in detail. Instead, HERG1 expression was found to regulate apoptosis in ESCC through thioredoxin domain-containing protein 5 (TXNDC5) by activating the phosphatidylinositol-3 kinase (PI3K)/Akt pathway (Wang et al., 2019). Yu et al. (2020) reported that the overexpression of the long non-coding RNA (lncRNA), heart and neural crest derivatives expressed 2 antisense RNA 1 (HAND2-AS1) promoted apoptosis in GC cells, and showed that it bound with miR-590-3p to alter the expression of potassium sodium-activated channel subfamily T member 2 (KCNT2). Potassium channel subfamily K member 9 (TASK-3) (*KCNK9* or K2P9.1) is a K^+^ channel from the K_2P_ family that forms functional homo- or heterodimers (Enyedi and Czirjak, 2010). Cikutović-Molina et al. (2019) recently showed that the knockdown of the TASK-3 gene promoted apoptosis in KATO III and MKN-45 human GC cell lines. The protein encoded by *potassium calcium-activated channel Subfamily M Alpha 1 (KCNMA1)* is a voltage- and Ca^2+^-activated K^+^ channel. Ma et al. (2017) found that *KCNMA1* significantly inhibited the biological malignant behavior of GC cells *in vitro* by inducing apoptosis, and suppressed xenograft tumor growth in subcutaneous mouse models. The importance of this study was to reveal that the anti-tumor effect of KCNMA1was mediated through suppressing the expression of the key apoptosis gene *protein tyrosine kinase 2 (PTK2)*.

**TABLE 1:** Overview of potassium channels with roles in the cell death and survival of upper gastrointestinal tract cancers.

**Channels**	**Organ**	**Mechanism/pathway**	**Induction**	**References**
KCNA5	GC		Aminopyridine, tetraethylammonium	Han et al., 2007
HERG	GC		Cisapride	Shao et al., 2005
	GC		siRNA technology	Shao et al., 2008
	GC		siRNA technology	Zhang et al., 2012
HERG1	ESCC	TXNDC5, PI3K/AKT pathway	shRNA technology	Wang et al., 2019
KCNT2	GC	HAND2-AS1, miR-590-3p	overexpression vector	Yu et al., 2020
KCNK9	GC		siRNA technology	Cikutović-Molina et al., 2019
KCNMA1	GC	PTK2	Overexpression plasmid	Ma et al., 2017

*ESCC, esophageal squamous cell carcinoma; GC, gastric cancer.*

Collectively, these findings clearly demonstrate the involvement of K^+^ channels in the apoptosis of ESCC and GC cells, and thus, further studies on their clinical potential are warranted.

### Chloride (Cl^–^) Channels

**FIGURE 1:**
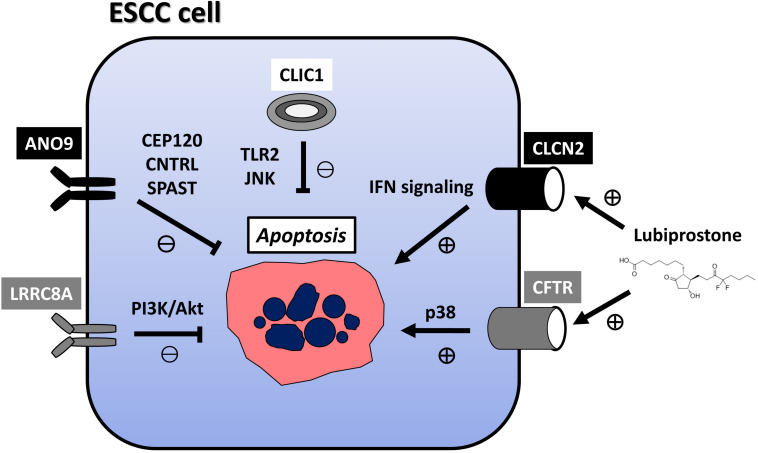
Mechanism of apoptosis regulation via Cl^−^ channels in ESCC cell. CLIC1 inhibits apoptosis via TLR2/JNK pathway. ANO9 inhibits apoptosis via centrosome-related genes, such as *CEP120*, *CNTRL*, and *SPAST*.LRRC8A inhibits apoptosis via PI3K/Akt signaling. CLCN2 induces apoptosis via IFN signaling. CFTR induces apoptosis in ESCC via activation of the p38 signaling pathway. Lubiprostone functions as activator of CLCN2 and CFTR, and increases apoptosis in ESCC cells. Lubiprostone is used in the management of idiopathic chronic constipation in patients with cancers, particularly those using opioid analgesics. ESCC: esophageal squamous cell carcinoma; CLIC1, chloride intracellular channel 1; ANO9, anoctamin 9; LRRC8A, leucine-rich repeat-containing protein 8A; CLCN2, chloride channel 2; CFTR, cystic fibrosis transmembrane conductance regulator.

Cl^–^ channels play a role in the cell death and survival of UGI cancers (Table 2). Ma et al. (2012) reported that the strong expression of chloride intracellular channel 1 (CLIC1) suppressed the proliferation of GC cells and promoted their apoptosis, migration, and invasion. Li et al. (2018) also demonstrated that the genetic knockdown of CLIC1 by siRNA technology strongly induced apoptosis in GC cells by regulating the mitogen-activated protein kinase (MAPK)/protein kinase B (Akt) pathways. Zhao et al. (2019) described a role for exosome-mediated transfer of CLIC1 in vincristine resistance via the regulation of P-gp and B-cell lymphoma 2 (Bcl-2) in GC cells. We previously reported the induction of apoptosis in ESCC via the Toll-like receptor 2 (TLR2)/c-Jun N-terminal kinase (JNK) pathway by the genetic knockdown of CLIC1 with siRNA technology (Kobayashi et al., 2018). The anoctamin family consists of transmembrane proteins in 10 isoforms, and the best-known anoctamin gene is *anoctamin 1* (*ANO1*), a Cl^–^ channel activated by Ca^2+^ (Schreiber et al., 2010). Seo et al. (2020) showed that 3n, Ani-FCC, a novel, potent, and selective ANO1 inhibitor, significantly enhanced apoptosis by activating caspase 3 and cleaving poly (ADP-ribose) polymerase (PARP) in GC cells. Xie et al. (2020) reported that long non-coding RNA (lncRNA) OPA-interacting protein 5 antisense transcript 1 (OIP5-AS1) regulated apoptosis in GC by targeting the microRNA (miR)-422a/ANO1 axis. We recently demonstrated that the genetic knockdown of ANO9 by siRNA technology increased apoptosis in ESCC cells (Katsurahara et al., 2020). Moreover, the findings of our microarray analysis indicated that the expression of a number of centrosome-related genes, such as centrosomal protein 120 (*CEP120*), *CNTRL*, and *SPAST*, was up- or down-regulated in ANO9-depleted KYSE150 cells, while immunohistochemistry (IHC) showed that the strong expression of ANO9 was associated with a poor prognosis in ESCC patients (Katsurahara et al., 2020). Over the past decade, one of the most important breakthroughs in cancer treatment has been immune checkpoint blockage (ICB) of programmed cell death-1 (PD-1). In GC, we have observed tumor suppressive effects following the genetic knockdown of ANO9 with siRNA technology, such as decreased proliferation, and increased apoptosis (Katsurahara et al., 2021). The results of microarray and IHC indicated that ANO9 regulates programmed cell death 1 ligand 2 (PD-L2) and binding ability to PD-1 via interferon (IFN)-related genes, suggesting that ANO9 has potential as a biomarker and target of ICB for GC. Leucine-rich repeat-containing protein 8A (LRRC8A) is a ubiquitous and integral component of the volume-regulated anion channel, which is required for the regulation of cell volume (Qiu et al., 2014). We reported that the depletion of LRRC8A promoted apoptosis in ESCC cells, microarray data revealed the altered regulation of phosphatidylinositol-3 kinase (PI3K)/Akt signaling in LRRC8A-depleted cells, and IHC showed that the strong LRRC8A expression correlated with a poorer prognosis in ESCC patients (Konishi et al., 2019). Chloride channel 2 (CLCN2) is a member of the CLC family, which is an inwardly rectifying chloride channel. We also demonstrated that downregulated expression of CLCN2 decreased apoptosis, whereas its upregulation increased it in ESCC cells (Mitsuda et al., 2021). The effects of lubiprostone, a CLCN2 activator, were also investigated, and apoptosis was increased in lubiprostone-treated ESCC cells. The results of microarray and IHC indicated that tumor progression is regulated by CLCN2 through its effects on IFN signaling, and that weak CLCN2 expression was associated with poorer outcomes in ESCC patients. Lubiprostone is used in the management of idiopathic chronic constipation in patients with various cancers, particularly those using opioid analgesics. Lubiprostone functioned as a pharmacological activator of CLCN2, and enhanced the inhibitory effects of cisplatin (CDDP) in ESCC cells (Mitsuda et al., 2021), suggesting the potential of its clinical application for ESCC. The cystic fibrosis transmembrane conductance regulator (CFTR) is a cyclic AMP-dependent chloride anion conducting channel, and inactivating germline mutations in CFTR cause the cystic-fibrosis (CF), which is the most common autosomal recessive hereditary disease among Caucasians. We have recently demonstrated that the overexpression of CFTR induced apoptosis in ESCC via activation of the p38 signaling pathway and was associated with a good patient prognosis (Matsumoto et al., 2021). The relationship between the incidence of cancer and genetic variations in the CFTR gene has been attracting increasing attention because CF patients are at a significantly higher risk of developing various cancers. Our results may explain the molecular mechanism of this clinical features and indicate the potential of CFTR as a mediator of and/or a biomarker for ESCC. Further, lubiprostone is also known as the CFTR activator, suggesting the future prospects of therapeutic strategies targeting CFTR against ESCC patients. Mechanism of apoptosis regulation via these Cl^–^ channels in ESCC cell were summarized in Figure 1.

**TABLE 2:** Overview of chloride channels with roles in the cell death and survival of upper gastrointestinal tract cancers.

**Channels**	**Organ**	**Mechanism/pathway**	**Induction**	**References**
CLIC1	ESCC	TLR2/JNK pathway	siRNA technology	Kobayashi et al., 2018
	GC		siRNA technology	Ma et al., 2012
	GC	PI3K/AKT, MAPK/ERK, and MAPK/p38 pathways	siRNA technology	Li et al., 2018
	GC	Exosome-mediated transfer	siRNA technology	Zhao et al., 2019
ANO1	GC		3n, Ani-FCC	Seo et al., 2020
	GC	LncRNA OIP5-AS1, miR-422a	RNA interference	Xie et al., 2020
ANO9	ESCC	Centrosome-related genes	siRNA technology	Katsurahara et al., 2020
	GC	IFN signaling, PD-L2	siRNA technology	Katsurahara et al., 2021
CLCN2	ESCC	IFN signaling	Lubiprostone, overexpression plasmid	Mitsuda et al., 2021
CFTR	ESCC	p38 signaling pathway	Overexpression plasmid	Matsumoto et al., 2021
LRRC8A	ESCC	Phosphatidylinositol 3-kinase/AKT signaling	siRNA technology	Konishi et al., 2019

*ESCC, esophageal squamous cell carcinoma; GC, gastric cancer.*

Collectively, these findings indicate the important roles of Cl^–^ channels in the apoptosis of UGI cancer cells and the potential of CLIC1, ANO1, ANO9, and LRRC8A as therapeutic targets against ESCC and GC.

### Calcium (Ca^2+^) Channels

Ca^2+^ channels participate in regulation of intracellular Ca^2+^ concentrations ([Ca^2+^]_i_), and play important roles in the cell death and survival of UGI cancers (Table 3). The transient receptor potential (TRP) superfamily comprises a very diverse group of ion channels, most of which exhibit permeability to monovalent and divalent cations. TRP family members are divided into seven subfamilies, such as classical (TRPC), vanilloid receptor-related (TRPV), and melastatin-related (TRPM) channels. Ding et al. (2018) previously demonstrated that pyrazolo[1,5-a]pyrimidine TRPC6 antagonists suppressed the proliferation of GC cells, and this anti-tumor effect on GC was confirmed in xenograft models using nude mice. We also recently reported that the genetic knockdown of TRPV2 by siRNA technology induced apoptosis in ESCC. A pathway analysis of microarray data showed that the depletion of TRPV2 down-regulated WNT/β-catenin signaling-related genes, and an IHC analysis revealed a correlation between strong TRPV2 expression and a poor prognosis in ESCC patients (Kudou et al., 2019). We also demonstrated the overexpression of TRPV2 in cancer stem cells (CSCs) derived from ESCC, and suggested the potential of tranilast, a TRPV2-specific inhibitor, as a therapeutic agent against CSCs (Shiozaki et al., 2018b). Tranilast has been used to treat patients with inflammatory diseases, such as asthma, dermatitis, allergic conjunctivitis, keloids, and hypertrophic scars, and its safety for clinical use has already been demonstrated. Preoperative adjuvant chemotherapy with CDDP and 5-fluorouracil (5FU) is currently used with beneficial effects to treat localized advanced ESCC in Japan (Ando et al., 2012). To confirm the clinical safety and efficacy of the additional use of tranilast with neoadjuvant 5-FU/CDDP, and to develop a novel therapeutic strategy for patients with advanced ESCC, we designed phase I/II study, and it is ongoing (Shiozaki et al., 2020). Chow et al. (2007) showed that TRPV6 mediated capsaicin-induced apoptosis in GC cells and also that capsaicin induced apoptosis by stabilizing p53 through the activation of JNK. Almasi et al. (2018) reported that the TRPM2 channel-mediated regulation of autophagy maintained mitochondrial function and promoted GC cell survival via the JNK signaling pathway. Quercetin induced apoptosis in GC cells by inhibiting MAPKs and TRPM7 channels (Kim et al., 2014). Calcium channel, voltage-dependent, alpha 2/delta subunit 3 (CACNA2D3) is an auxiliary member of the alpha-2/delta subunit family of the voltage-dependent Ca^2+^ channel complex. Li et al. (2013) reported the down-regulation of CACNA2D3 in 56.7% of ESCC, which correlated with lymph node metastasis, TNM staging, and the poor outcomes of ESCC patients, and suggested that CACNA2D3 up-regulates intracellular cytosolic Ca^2+^, thereby inducing apoptosis. Nie et al. (2019) demonstrated that CACNA2D3 enhanced the chemosensitivity of ESCC to cisplatin by inducing Ca^2+^-mediated apoptosis and suppressing the PI3K/Akt pathways. Recently, we have demonstrated that voltage-gated Ca^2+^ channels (VGCCs), including calcium voltage-gated channel auxiliary subunit alpha2delta 1 (CACNA2D1) and calcium voltage-gated channel auxiliary subunit beta 4 (CACNB4), were strongly expressed in gastric CSCs (Shiozaki et al., 2021). The cytotoxicities of the CACNA2D1 inhibitor amlodipine and the CACNB4 inhibitor verapamil were greater at lower concentrations in CSCs than in non-CSCs. Amlodipine, a specific inhibitor of CACNA2D1, has been widely used in the treatment of hypertension and cardiac angina. Verapamil, a specific inhibitor of CACNB4, has been widely used in the treatment of arrhythmia. These results indicate that VGCCs play a role in maintaining CSCs, and demonstrated the potential of their specific inhibitors as targeted therapeutic agents against GC.

**TABLE 3:** Overview of calcium channels with roles in the cell death and survival of upper gastrointestinal tract cancers.

**Channels**	**Organ**	**Mechanism/pathway**	**Induction**	**References**
TRPC6	GC		Pyrazolo[1,5-a]pyrimidine	Ding et al., 2018
TRPV2	ESCC	WNT/β-catenin signaling, Cancer stem cells	Tranilast, siRNA technology	Kudou et al., 2019
TRPV6	GC	Bax, p53, JNK	Capsaicin, overexpression plasmid	Chow et al., 2007
TRPM2	GC	JNK signaling pathway	shRNA technology	Almasi et al., 2018
TRPM7	GC	MAPK	Quercetin	Kim et al., 2014
CACNA2D1	GC	Cancer stem cells	Amlodipine	Shiozaki et al., 2021
CACNA2D3	ESCC	Up-regulate intracellular free cytosolic Ca^2+^	Overexpression vector	Li et al., 2013
	ESCC	Ca^2+^-mediated apoptosis, PI3K/Akt pathways	Cisplatin, overexpression plasmid	Nie et al., 2019
CACNB4	GC	Cancer stem cells	Verapamil	Shiozaki et al., 2021

*ESCC, esophageal squamous cell carcinoma; GC, gastric cancer.*

These findings clearly demonstrate that Ca^2+^ channels contribute to UGI cancer cell death, and thus, future studies are needed on their clinical potential.

### Water Channels

Under physiological and pathophysiological conditions, the volume of cells is regulated and the electrolyte balance is maintained by aquaporins (AQPs), which are transmembrane proteins that facilitate water transport. Thirteen AQP subtypes have so far been identified in humans and their functions have been elucidated. AQPs have also been shown to play important roles in the cell death and survival of UGI cancers (Table 4). We recently demonstrated the induction of apoptosis in ESCC cells by the genetic knockdown of AQP1 with siRNA technology, alterations in Death receptor signaling pathway-related genes in AQP1-depleted TE5 cells by a microarray analysis, and a correlation between the cytoplasmic dominant expression of AQP1 and a poor prognosis in patients with ESCC by IHC (Yamazato et al., 2018). Sun et al. (2016) reported that AQP1 expression in GC was associated with apoptosis and the survival of patients, however, molecular mechanisms of the regulation of apoptosis via AQP1 had not been shown in detail. Kusayama et al. (2011) attributed cell death in ESCC due to the genetic knockdown of AQP3 by siRNA technology to the direct interference of cell adhesion involving the intracellular focal adhesion kinase (FAK)-MAPK signaling pathways. Several studies demonstrating the regulatory mechanisms via miR were valuable. Jiang et al. (2014) demonstrated that the down-regulation of Bcl-2 and up-regulation of caspase-3 activity and Bcl-2-associated X protein (Bax) were involved in the induction of cell apoptosis in GC cells by miR-874 through the targeting of AQP3. Furthermore, Zhu et al. (2020) showed that the up-regulated expression of miR-877 promoted apoptosis in GC cells, and luciferase reporter assays revealed that AQP3 was a direct downstream target of miR-877. We also demonstrated that the genetic knockdown of AQP5 by siRNA technology induced apoptosis in ESCC cells, while a microarray analysis identified tumor protein p53-induced nuclear protein 1 (TP53INP1) as one of the top ranking up-regulated genes widely known as the gene related to cell apoptosis in AQP5-depleted ESCC cells (Shimizu et al., 2014). IHC staining of samples collected from ESCC patients revealed relationships between the expression of AQP5 and tumor size, histological type, and tumor recurrence (Shimizu et al., 2014).

**TABLE 4:** Overview of water channels with roles in the cell death and survival of upper gastrointestinal tract cancers.

**Channels**	**Organ**	**Mechanism/pathway**	**Induction**	**References**
AQP1	ESCC	Death receptor signaling pathway	siRNA technology	Yamazato et al., 2018
	GC	Apoptosis		Sun et al., 2016
AQP3	ESCC	FAK-MAPK signaling pathways	pan-AQP inhibitor, siRNA technology	Kusayama et al., 2011
	GC	miR-874	Overexpression vector	Jiang et al., 2014
	GC	miR-877	miRNA mimics	Zhu et al., 2020
AQP5	ESCC	p53	siRNA technology	Shimizu et al., 2014

*ESCC, esophageal squamous cell carcinoma; GC, gastric cancer.*

Therefore, AQPs appear to be crucially involved in the cell death and survival of ESCC and GC, and these findings indicate the potential of AQP1, 3, and 5 as therapeutic targets in UGI cancers.

### pH Regulators

The regulation of cytoplasmic pH by ion transporters was recently shown to be important for tumor cell functions. pH regulators, including the anion exchanger (AE), Na^+^/H^+^ exchanger (NHE), and vacuolar H^+^-ATPase (V-ATPase), directly contribute to maintaining the tumor microenvironment, and play critical roles in the cell death and survival of UGI cancers (Table 5). The electroneutral exchange of Cl^–^ for HCO_3_^–^ across the plasma membrane of mammalian cells is facilitated by AE proteins and maintains intracellular pH. Three AE isoforms have been identified: AE1, AE2, and AE3. Shen et al. (2007) showed that the genetic knockdown of AE1 by siRNA technology in GC cells induced the release of p16 from the cytoplasm to the nucleus, leading to the death of tumor cells. Importantly, the transfection with miR-24 induced the return of p16 to the nucleus, confirming the miR-24-controlled AE1 down-regulation in GC (Wu et al., 2010). We performed IHC on primary tumor samples obtained from ESCC patients and found that significantly fewer samples exhibited diffuse AE1 expression than focal expression (Shiozaki et al., 2017). In addition, the genetic knockdown of AE1 by siRNA technology induced apoptosis, and a microarray analysis of AE1-depleted ESCC cells showed the down-regulated expression of MAPK and Hedgehog signaling pathway-related genes (Shiozaki et al., 2017). However, we subsequently demonstrated that the depletion of AE2 in ESCC cells enhanced cell migration and suppressed the induction of apoptosis (Shiozaki et al., 2018a). The microarray analysis of AE2-depleted ESCC cells also showed the changes expression of various matrix met alloproteinase (MMP) signaling pathway-related genes. The expression levels of MMP1 and MMP12 mRNA were higher, and mRNA of tissue inhibitor of metalloproteinase 4 (TIMP4), metalloproteinase inhibitor, was lower in AE2-depleted ESCC cells, suggesting that MMP regulation is a key mechanism by which AE2 controls the movement of ESCC cells. Further, IHC staining revealed a relationship between the weak expression of AE2 at the invasive front and shorter postoperative survival in ESCC patients (Shiozaki et al., 2018a).

**TABLE 5:** Overview of pH regulators with roles in the cell death and survival of upper gastrointestinal tract cancers.

**Channels**	**Organ**	**Mechanism/pathway**	**Induction**	**References**
AE1	ESCC	MAPK and Hedgehog signaling pathways	siRNA technology	Shiozaki et al., 2017
	GC	p16	siRNA technology	Shen et al., 2007
AE2	ESCC		siRNA technology	Shiozaki et al., 2018a
NHE1	EAC		Deoxycholic acid	Goldman et al., 2011
	EAC		Amiloride, Guggulsterone	Guan et al., 2014
	ESCC	PI3K-AKT signaling, Notch signaling	siRNA technology	Ariyoshi et al., 2017
	GC		Antisense gene	Liu et al., 2008
V-ATPase	GC		Proton pump inhibitors	Chen et al., 2009
	GC	Phosphorylation of LRP6, Wnt/β-catenin signaling	Diphyllin	Shen et al., 2011
	GC		Pantoprazole	Shen et al., 2013

*ESCC, esophageal squamous cell carcinoma; EAC, esophageal adenocarcinoma; GC, gastric cancer.*

NHE plays a role in the regulation of intracellular pH by mediating the coupled counter-transport of one H^+^ for one Na^+^. Goldman et al. (2011) demonstrated that NHE controlled deoxycholic acid-induced apoptosis in EAC cells via a H^+^-activated, Na^+^-dependent ionic shift. Guan et al. (2014) reported the strong expression of NHE1 in EAC tissues, and showed that the knockdown of NHE1 in EC cells reduced their viability and induced apoptosis. We also demonstrated the inhibition of apoptosis and activation of PI3K/Akt signaling in ESCC cells following the knockdown of NHE1 (Ariyoshi et al., 2017) in ESCC. These findings indicated that the Notch signaling pathway was inhibited by the knockdown of NHE1, while IHC of primary ESCC samples showed a lower survival rate in the NHE1 low group than in the NHE1 high group (Ariyoshi et al., 2017). Liu et al. (2008) reported the significant suppression of the malignant behavior of human GC cells, the inhibition of cell growth, the induction of cell apoptosis, and the partial reversal of the malignant phenotypes of SGC-7901 following NHE1 antisense gene transfection.

V-ATPase is a proton pump in cells that is important for the maintenance of intracellular pH. Chen et al. (2009) showed that proton pump inhibitors (PPIs) reduced intracellular pH in SGC7901, human GC cell line, by suppressing V-ATPase and also promoted the apoptotic effects of antitumor drug, adriamycin. They showed that administration of adriamycin after PPI pretreatment produced the most cytotoxic effects on SGC7901 cells and increased the early and total apoptosis rates. PPI also significantly reduced the adriamycin-releasing index and increased the intracellular adriamycin concentration. Shen et al. (2011) demonstrated that diphyllin, a new V-ATPase inhibitor, decreased intracellular pH and induced apoptosis by inhibiting the phosphorylation of low-density lipoprotein receptor-related protein 6 (LRP6) in Wnt/β-catenin signaling. They subsequently reported that pantoprazole induced the apoptosis of GC cells, and indicated the potential of pantoprazole as a V-ATPase inhibitor for the treatment of GC through the suppressed phosphorylation of LRP6 in Wnt/β-catenin signaling (Shen et al., 2013).

Collectively, these findings indicate the potential of pH regulators, including AEs, NHEs, and V-ATPases, as key therapeutic targets, and the silencing of their expression may represent a novel therapeutic strategy against UGI cancers.

## Conclusion

There is recent considerable emphasis on determining better treatment strategies for advanced cancers with poor prognosis. UGI cancers are aggressive, rapidly metastasize and still have low survival rate, especially for ESCC. Further, disseminated metastasis is one of the most common forms of recurrence of disease and is associated with a poor prognosis in GC patients. Therefore, detecting effective targets for UGI cancers is crucial for improving treatment options. Current information on advances in cellular physiological research on the roles of ion and water channels/transporters in the cell death and survival of UGI cancers was systematically reviewed herein. Human EC and GC express many different ion channels, AQPs, and pH regulators, the pharmacological manipulation and gene silencing of which affected apoptosis, and may be involved in tumorigenesis and progression. The findings of previous studies indicate the roles of ion transporter, water channels, and pH regulators as functional biomarkers and therapeutic targets in EC and GC. One of the powerful charms of the research in this field is that anti-cancer effect has been newly identified in several inhibitors or stimulators of ion channels, such as lubiprostone, tranilast, amlodipine, verapamil, and PPI, which have been used to treat patients with other disorders, and their safeties for clinical use has already been demonstrated. In addition, the targeting of ion and water channels specifically activated in CSCs may become an important strategy for cancer therapy. On the other hand, there are inborn limitations of the review, such as the lack of a critical view of these channels in the current clinical oncology field. A more detailed understanding of the molecular mechanisms regulating cell death and survival may result in the application of cellular physiological strategies, such as the regulation of ion transporters, water channels, and intracellular pH, as novel therapeutic approaches for UGI cancers.

## Author Contributions

AS wrote the manuscript. YM and EO edited the manuscript. All authors contributed to the article and approved the submitted version.

## Conflict of Interest

The authors declare that the research was conducted in the absence of any commercial or financial relationships that could be construed as a potential conflict of interest.
